# How Can the Evidence from Global Large-scale Clinical Trials for Cardiovascular Diseases be Improved?

**DOI:** 10.1186/1756-0500-4-222

**Published:** 2011-06-29

**Authors:** Hiroshi Sawata, Kiichiro Tsutani

**Affiliations:** 1 Department of Drug Policy and Management, Graduate School of Pharmaceutical Sciences, The University of Tokyo, 7-3-1, Hongo, Bunkyo-ku, Tokyo, Japan

## Abstract

**Background:**

Clinical investigations are important for obtaining evidence to improve medical treatment. Large-scale clinical trials with thousands of participants are particularly important for this purpose in cardiovascular diseases. Conducting large-scale clinical trials entails high research costs. This study sought to investigate global trends in large-scale clinical trials in cardiovascular diseases.

**Findings:**

We searched for trials using clinicaltrials.gov (URL: http://www.clinicaltrials.gov/) using the key words 'cardio' and 'event' in all fields on 10 April, 2010. We then selected trials with 300 or more participants examining cardiovascular diseases. The search revealed 344 trials that met our criteria. Of 344 trials, 71% were randomized controlled trials, 15% involved more than 10,000 participants, and 59% were funded by industry. In RCTs whose results were disclosed, 55% of industry-funded trials and 25% of non-industry funded trials reported statistically significant superiority over control (p = 0.012, 2-sided Fisher's exact test).

**Conclusions:**

Our findings highlighted concerns regarding potential bias related to funding sources, and that researchers should be aware of the importance of trial information disclosures and conflicts of interest. We should keep considering management and training regarding information disclosures and conflicts of interest for researchers. This could lead to better clinical evidence and further improvements in the development of medical treatment worldwide.

## Background

The evidence from a large number of clinical trials can be beneficial for the medical practice of many health care providers, informing clinical practice guidelines by providing a rationale for determining the most appropriate treatment. Accordingly, this evidence can be also beneficial for patients by receiving the most appropriate treatment.

Clinical trials are critical for obtaining new clinical evidence. In cardiovascular diseases, large-scale clinical trials involving thousands of participants are particularly important for evaluating the risk reductions of cardiac events and/or death, because of the requirement for the evaluation of cardiovascular events with relatively small differences in incidences between groups. These clinical trials can provide important evidence about the most appropriate treatment regimen for preventing cardiovascular and metabolic diseases. In the 1970s, researchers began to conduct large-scale clinical trials in Western countries with the incidence of cardiovascular events as endpoints [1,2]. The number of large-scale clinical trials has markedly increased since then. Recently, a large number of clinical trials for evaluating the incidence of cardiac events and/or death using hard endpoints have been conducted in cardiovascular and metabolic medicine.

We reviewed important issues surrounding large-scale clinical trials in Japan from 2007 [3,4]. The major issues revealed by the review were the funding sources and infrastructure surrounding of clinical trials. The review indicated that financial and infrastructural resources must be maintained to conduct clinical trials appropriately. However, high research costs are involved in clinical trials. Currently, clinical trials receive funding from various sources, including public agencies, private companies, foundations, etc. It has been proposed that industry-funded trials may be more likely to produce biased results, interpretations and conclusions [5-11]. Ridker, et al. reported that cardiovascular trials reported between 2000 and 2005 funded by for-profit organizations were more likely to report positive findings than those funded by not-for-profit organizations [5].

Considering this situation, it is important to clarify the problems and solutions surrounding current large-scale clinical trials. Thus, in this study we sought to elucidate the current situation and important global trends in large-scale clinical trials examining cardiovascular diseases.

## Methods

We searched for trials using clinicaltrials.gov (URL: http://www.clinicaltrials.gov/) with the words 'cardio' or 'event' in all fields on 10 April 2010. We then selected all large-scale clinical trials examining cardiovascular diseases. We defined 'large-scale clinical trials' as trials involving 300 participants or more. If the trial was on-going and the planned number of participants was 300 or more, this trial was regarded as a 'large-scale clinical trial'. We defined 'trials examining cardiovascular diseases' as trials where the primary endpoint was the incidence of cardiovascular events, e.g. myocardial infarction, chronic heart failure, ischemic heart attack, and/or mortality including death.

For the target trials, we recorded 11 items: 1) primary objective of trial, 2) trial design, 3) interventions, 4) sponsor(s), 5) starting year and month, 6) ending year and month, 7) number of enrolled participants or target number of participants, 8) countries of trial sites, 9) results of trial, 10) publications linked from clinicaltrials.gov, 11) funding sources.

We categorized the trials by each criterion, and calculated summary statistics. The relationship between funding sources (industry or non-industry) and the results of a primary analysis (positive or negative) were tested using 2-sided Fisher's exact tests. A result was regarded as positive if a trial met the primary objective of statistically significant superiority or non-inferiority over control conditions or behavioral interventions.

## Results

### Screening of large-scale clinical trials in cardiovascular diseases

**Figure 1:**
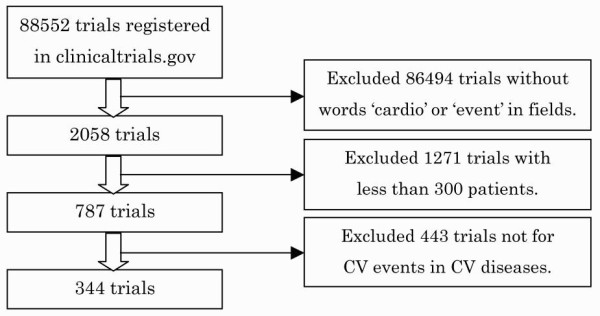
**Screening of trials**. CV: cardiovascular

We showed the number of trials during screening of the trials in Figure 1. We found 2,058 trials registered in clinicaltrials.gov with the words 'cardio' or 'event' in all fields as of 10 April, 2010. Of 2,058 trials, 787 met the criterion of involving 300 participants or more. Of these 787 trials, 344 met the criteria that the primary endpoint of the trial was the incidence of cardiovascular events and the trial population was patients with cardiovascular diseases.

None of these 344 trials were excluded from our analysis because of insufficient information.

### Trial design (RCT/non-RCTs) and number of participants

We categorized all 344 trials according to the trial design and the number of participants (Table 1). Of these trials, 71% (243/344) were randomized controlled trials (RCTs), and 29% (101/344) were not RCTs (non-RCTs). Examining the number of participants revealed that 0.6% (2/344) of the trials involved 100,000 participants or more, 2% (6/344) of trials involved between 30,000 and 99,999 participants, 12% (42/344) involved between 10,000 and 29,999 participants, and 35% (121/344) of trials involved less than 1,000 participants. The results revealed a trend towards an association between smaller proportion of trials and larger numbers of participants. The statistical significance of this trend was not tested because of small sample sizes.

**Table 1:** Numbers of trials by number of participants and trial design (RCT or Non-RCT)

Number of participants	Number of trials				Proportion of RCT (%)
		
	RCT	Non-RCT	Total		
≥100,000	0	2	2	(0.6%)	0
30,000-99,999	2	4	6	(1.7%)	33.3
10,000-29,999	37	5	42	(12.2%)	88.1
3,000-9,999	56	12	68	(19.8%)	82.4
1,000-2,999	72	33	105	(30.5%)	68.6
< 999	76	45	121	(35.1%)	62.8

Total	243	101	344	(100%)	70.6

RCT: randomized controlled trial; Non-RCT: non-randomized controlled trial

In trials involving between 3,000 and 9,999 participants, and those involving between 10,000 and 29,999 participants, the proportions of RCTs were 82% and 88%, respectively. In trials involving less than 1,000 participants, and those involving between 1,000 and 2,999 participants, the proportion that were RCTs was 63% and 69%, respectively. These proportions were substantially lower for trials involving between 3,000 and 29,999 participants.

### Number of trials by starting year

**Figure 2:**
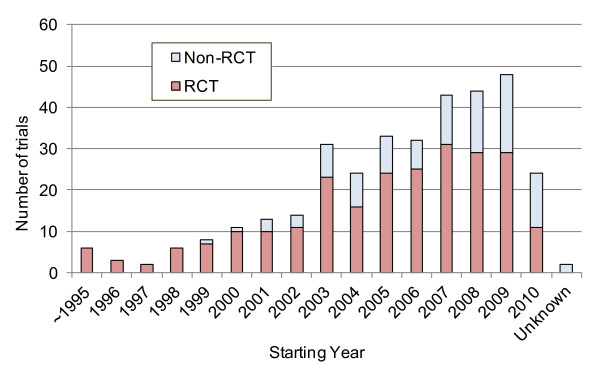
**Trends in the number of trials by starting year and trial design (RCT or non-RCT)**.

We categorized the numbers of trials according to the starting year (Figure 2). The number of large-scale clinical trials increased gradually from the latter 1990s, and showed a 2.4-fold increase in 2003 relative to 2002. Fifty-nine trials were registered with clinicaltrials.gov in 2009. This was the highest number of all the years we examined. It should be noted that the number of trials reported for 2010 includes trials registered before 10 April, 2010.

A larger proportion of trials were RCTs before 2000, relative to after 2000. Conversely, the number of non-RCTs increased after 2000. From 2005, the trend of the increasing number of non-RCTs became more obvious, although number of RCTs has not changed noticeably during this period.

### Number of trials by funding sources

We categorized trials according to the category of funding sources; industry, academic, government, foundation, and other. We found that 204 trials (59%) were funded by industry (e.g. pharmaceutical companies or medical device providers), 122 trials (35%) were funded by academic organizations, e.g. universities and research centers, 55 trials (16%) were funded by governmental organizations, 32 trials (9%) were funded by foundations, 48 trials (14%) were funded by other organizations (e.g. hospitals and individuals), 97 trials (28%) were funded by multiple funding organizations, and 17 trials (5%) were funded by both governmental organizations plus industries or foundations (Table 2).

**Table 2:** Numbers of trials by funding source and trial design (RCT or non-RCT)

Funding source	Number of trials				Proportion of RCT (%)
		
	RCT	Non-RCT	Total		
Industry	142	62	204	(59.3%)	69.6
Academic	79	43	122	(35.5%)	64.8
Government	44	11	55	(16.0%)	80.0
Foundation	28	4	32	(9.3%)	87.5
Other	37	11	48	(14.0%)	77.1

Total	243	101	344	(100%)	70.6

**Figure 3:**
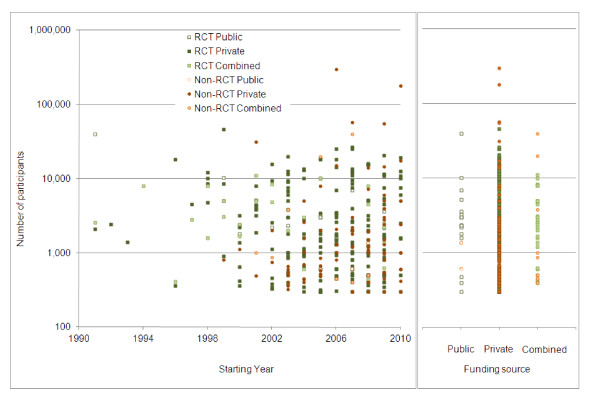
**Dot plot by starting year, funding source, and number of participants**.

We analyzed the relationship between starting years of trials, types of funding sources, and the numbers of participants (Figure 3). 'Public' funding sources included governmental organizations, and 'Private' sources included industry, academic institutions, foundations, and others.

Trials with larger numbers of participants were mainly conducted with funding from private sources.

Summary statistics of the numbers of participants according to funding sources are shown in Table 3. The median of number of participants in publicly funded trials was 2,331, and that in trials with combined funding sources was 2,450. The median number of participants in privately funded trials was 1,400, substantially lower than publicly funded trials and trials with combined funding sources.

**Table 3:** Summary statistics regarding numbers of participants by funding source

Parameter	Public	Private	Combined	Total
Number of trials (N)	19	289	36	344

Number of participants				
≥100,000	0	2	0	2
30,000-99,999	1	4	1	6
10,000-29,999	1	38	3	42
3,000-9,999	6	51	11	68
1,000-2,999	6	88	11	105
< 999	5	106	10	121

Mean	4,696	5,944	4,703	5,745
Standard deviation	8,877	21,485	7,299	19,934
Median	2,331	1,400	2,450	1,555
Max	39,876	300,000	40,000	300,000

### Number of trials by region or country

We calculated the number of trials with multiple participating countries according to the starting year of trials (Table 4). The results showed that 227 trials (68%) were conducted in a single country, while 106 trials (32%) were conducted in multiple countries. The number of trials conducted in single country showed a 7.1-fold increase between 2006 and 2010 compared with those conducted before 2000, although those conducted in multiple countries showed a 3.1-fold increase by the 2006-2010 period compared with those conducted before 2000. The number of trials involving more than 20 countries from 2001-2005 and from 2006-2010 was 16 and 17, respectively, and one trial involving more than 20 countries was conducted before 2000.

**Table 4:** Number of participating countries by starting year of trials

Number of countries	Number of trials (N = 333*)				
	
	-2000	2001-2005	2006-2010	Total	
Single country	19	73	135	227	(68.2%)

Multiple countries	16	40	50	106	(31.8%)
2-5 countries	8	14	13	35	(10.5%)
6-20 countries	7	10	20	37	(11.1%)
> 20 countries	1	16	17	34	(10.2%)

* 11 trials whose starting years or countries were unknown were excluded.

We investigated the regions involved and starting year in each trial. 171 trials (51%) were in Western Europe, which was the most frequently involved region. 161 trials (48%) were conducted in North America, 95 trials (28%) were in East Asia. Only 32 trials (10%) were in Africa and 33 (10%) were in Southeast Asia. Until 2000, North America and Western Europe were the only major regions involved in large-scale clinical trials. Between 2006 and 2010, however, East Asia became a major region of involvement.

### Relationship between results and funding sources

In 83 (24%) RCTs whose results were disclosed, we counted the number of trials according to the primary result and funding source. Trial results with parallel group comparisons were grouped into two categories, positive and negative, according to the statistical superiority or non-inferiority relative to control, revealed by primary analysis.

Thirty of 55 (55%) industry-funded trials and seven of 28 (25%) non-industry funded trials showed statistical superiority or non-inferiority to control. Forty-six (55%) trials failed to meet the primary objective. The relationship between trial results and funding sources was statistically significant (p = 0.012, 2-sided Fisher's exact test).

## Discussion

We found 344 trials involving 300 or more participants that were registered with clinicaltrials.gov, using the search terms 'cardio' and 'event' in any field. All of the selected trials were conducted to evaluate the incidence of cardiovascular events and/or death in cardiovascular diseases.

Of 344 trials, more than 70% were RCTs, and 15% of trials enrolled more than 10,000 participants. In trials involving between 3,000 and 9,999 participants, and those involving between 10,000 and 29,999 participants, the proportion of trials that were RCTs was greater than 0.8, substantially higher than the proportion of RCTs among trials involving less than 3,000 participants.

We categorized the number of trials by starting year. The number of large-scale clinical trials increased gradually from the latter 1990s, and showed 2.4-fold increase in 2003 compared with 2002. From 2003, number of non-RCTs increased obviously. This increase was considered to be related to the International Committee of Medical Journal Editors' (ICMJE) request for the registration of trials that began enrolling participants any time before July 1, 2005, if investigators wished to submit the results of their trial to journals that follow ICMJE policy [12,13]. This requirement might have increased investigators' awareness of the importance of trial registration. Fifty-nine trials registered with clinicaltrials.gov started in 2009. It should be noted that the number of trials reported for 2010 includes those registered before 10 April, 2010. The number of trials registered with clinicaltrials.gov has thus steadily increased.

Regarding funding sources, about 60% of trials were funded by private industry, e.g. pharmaceutical companies or medical device providers, 28% were funded by multiple funding organizations, and 5% were funded by a combination of governmental organizations and industries or foundations. These results revealed that various organizations funded large-scale clinical trials, and sponsors sometimes required multiple funding sources to conduct large-scale clinical trials. When we examined the 50 trials with 10,000 participants or more, 40 trials (80%) were found to be funded by industry (or industry plus other organizations), while six trials (12%) were funded at least partially by governmental organizations, and only two trials (4%), both started in the 1990s in the United States, were solely funded by governmental organizations such as the National Heart, Lung, and Blood Institute (NHLBI).

Our examination of the relationship between the types of funding sources and participant numbers revealed that the median number of participants involved in privately funded trials was lower than that involved in publicly funded trials and trials with combined funding. On the other hand, the mean and maximum of numbers of privately funded trials were higher than for publicly funded trials and trials with combined funding sources. Trials with larger numbers of participants were mainly conducted with funding from private sources, although there were also a large number of smaller trials funded by private sources. These findings indicate that it is relatively difficult to conduct large-scale clinical trials funded solely by governmental organizations.

According to the records in clinicaltrials.gov, 26 of 344 trials (8%) did not disclose their planned ending years. Of 308 trials whose ending years were disclosed, 127 trials have been completed by the end of 2009. However, in September 2010, the results of only 77 trials (61%) commenced before 2009 were accessible, and the results of 50 trials (39%) were not disclosed. In other words, we could not access to the ending years or results of 76 trials (22%). This result indicates that some sponsors did not update the registered information in clinicaltrials.gov.

In 83 RCTs where publications were disclosed, 55% of industry-funded trials and only 25% of non-industry funded trials reported statistically significant superiority of the target intervention relative to control. This difference was statistically significant (p = 0.012, 2-sided Fisher's exact test), indicating that industry-funded trials were significantly more likely to report statistically significant differences favoring target interventions compared with references. This result is consistent with the findings of previous reports [5-11], showing that industry-funded trials tended to produce results in favor of target interventions compared with references.

It should be noted that more than half of the trials failed to meet the primary objective. This means not only sacrifices in terms of the voluntary cooperation of participants, but also represents the wasting of limited resources. This finding suggests that sponsors should discuss the rationale of their study design and sample size in detail before trials begin. Conducting small pilot trials may provide an appropriate solution for avoiding unsuccessful trials. The involvement of independent data monitoring committees, including third party biostatisticians, may be another worthwhile solution. In addition, sponsors should stop or modify the trial when blinded interim analysis indicates that there is a strong possibility the primary objective will not be achieved. A greater number of worthwhile trials will be possible if the waste of resources by failed trials is reduced.

The current study suffers from the limitation that all data were obtained from clinicaltrials.gov, which is run by NIH in United States. As such, clinical trials in which the United States was not involved may have been omitted.

## Conclusions

Our research revealed that number of large-scale clinical trials in cardiovascular diseases increased steadily over time, and that clinical trials are becoming increasingly globalized. This indicates that the quality and quantity of evidence is improving. However, sponsors should aware that the timely update of the registered trial information in clinicaltrials.gov is important.

Our findings highlight a concern about the potential bias related to funding sources. More than 80% of the trials we found were conducted by private funding sources, especially clinical trials with larger numbers of participants. Therefore, sponsors as well as researchers at each site should be aware of the importance of conflicts of interest. The ICMJE requests that sponsors disclose certain information regarding the trial management, including funding sources. We should keep considering management and training regarding conflicts of interest. Sponsors should also be aware that minimizing the waste of resources by careful consideration of their study designs is also one of their responsibilities as sponsor.

Addressing these issues may facilitate the improvement of the quality of clinical evidence, and the development of better medical treatment worldwide.

## Competing interests

HS is an employee of Novartis Pharma K.K.. The Department of Drug Policy and Management, Graduate School of Pharmaceutical Sciences, The University of Tokyo, is endowed department by Towa Pharmaceutical Co., Ltd., one of the leading companies of generic drugs in Japan.

## Authors' contributions

HS examined the study and drafted the manuscript. KT helped to draft the manuscript. All authors read and approved the final manuscript.
